# Virtual Distances Methodology as Verification Technique for AACMMs with a Capacitive Sensor Based Indexed Metrology Platform

**DOI:** 10.3390/s16111940

**Published:** 2016-11-18

**Authors:** Raquel Acero, Jorge Santolaria, Agustin Brau, Marcos Pueo

**Affiliations:** 1 Centro Universitario de la Defensa, Academia General Militar, Ctra. Huesca s/n, Zaragoza 50090, Spain; mpueo@unizar.es; 2 Department of Design and Manufacturing Engineering, University of Zaragoza, María de Luna 3, Zaragoza 50018, Spain; jsmazo@unizar.es; 3 Department of Industrial Engineering, University of Sonora, Rosales y Blvd. Luis Encinas s/n, Hermosillo 83000, Sonora, Mexico; agustin.brau@unison.mx

**Keywords:** AACMM, indexed metrology platform, virtual distance, verification

## Abstract

This paper presents a new verification procedure for articulated arm coordinate measuring machines (AACMMs) together with a capacitive sensor-based indexed metrology platform (IMP) based on the generation of virtual reference distances. The novelty of this procedure lays on the possibility of creating virtual points, virtual gauges and virtual distances through the indexed metrology platform’s mathematical model taking as a reference the measurements of a ball bar gauge located in a fixed position of the instrument’s working volume. The measurements are carried out with the AACMM assembled on the IMP from the six rotating positions of the platform. In this way, an unlimited number and types of reference distances could be created without the need of using a physical gauge, therefore optimizing the testing time, the number of gauge positions and the space needed in the calibration and verification procedures. Four evaluation methods are presented to assess the volumetric performance of the AACMM. The results obtained proved the suitability of the virtual distances methodology as an alternative procedure for verification of AACMMs using the indexed metrology platform.

## 1. Introduction

Calibration and verification procedures for portable coordinate measuring machines use calibrated gauges that establish the reference lengths that should be used to calculate the instrument’s measurement error. Articulated arm coordinate measuring machines (AACMMs) inherited the use of reference gauges from the verification processes carried out for coordinate measuring machines (CMMs), where the verification tries to reproduce the real measuring process by using calibrated objects. Various reference artifacts are used in CMMs calibration and verification processes, with the main target being to minimize the artifact’s testing poses [1,2,3]. One-dimensional gauges, such as ball bars or calibrated blocks, two-dimensional targets, such as sphere plate gauges, and three-dimensional targets, such as spheres, cubes or tetrahedra, are commonly used for this purpose. Multi-dimensional gauges help reduce the number of testing positions required for the reference artifact during setup, thereby decreasing the time and cost of these procedures. One-dimensional gauges are widely used in verification procedures due to their flexibility of positioning and reasonable costs compared to more complex gauges. For AACMMs, it is important to consider the maximum measurement range of the arm to select the adequate portable gauges, which are placed in the poses defined in the evaluation standards. The applicable standards for evaluating AACMMs are the ASME B89.4.22-2004 standard [4], the VDI/VDE 2617-2009 part 9 guideline [5] and the draft of the ISO/CD 10360 part 12-2014 [6]. These guidelines allow the metrological characterization of the arm in terms of its volumetric accuracy and point repeatability using calibrated reference artifacts. They are set in multiple poses of the arm’s working volume and materialize the reference length to calculate the error from the arm in the measuring process.

The development of new reference artifacts and techniques for the verification and calibration of AACMMs is an active research field. As mentioned, one-dimensional gauges are the most common because of their easy use, flexibility of positioning, high accuracy and low cost compared to other types of gauges. Kovac and Frank [7] develop a new high-precision measuring device for AACMM testing and calibration based on laser interferometer measurements along a line gauge beam. Sladek et al. [8] establish a virtual simulation system called a virtual articulated arm, which evaluates the measurement accuracy and generates a compensation matrix using linear gauges. Santolaria et al. [9,10,11,12,13] report on new calibration methods for measuring arms by using ball bar gauges for multiple positions and orientations of the arm’s working volume. Using three-dimensional reference artifacts, Shimojima et al. [14] present a method for estimating the uncertainty of a measuring arm by using a ball plate gauge with nine spheres located at different heights on a metal plate surface, which is measured with the arm at five positions and orientations. Then, the distances between the centers of the measured spheres are compared to the nominal distances obtained using a CMM to evaluate the measurement performance of the arm. A similar approach using three-dimensional gauges is described in [15]. The use of kinematic seats in the calibration procedures of AACMMs is also common and has numerous advantages, as shown in [16], where the single point repeatability of the arm is estimated. Additionally, Gatti et al. [17] propose a kinematic seat plate as a reference artifact for AACMM calibration procedures. Piratelli [18] introduces the development of a virtual geometry gauge called a virtual ball bar to evaluate the performance of AACMMs. The gauge has two groups of four holes on each side of the bar, which determines points of the spherical surfaces. These points are fitted to spheres, and the distances between the spheres’ centers are calculated and compared to the calibrated length. In further works from the same author [19,20], a virtual sphere plate gauge is developed defining 16 groups of four conical holes placed on aluminum pyramidal blocks. These groups determine 16 virtual spheres by taking points in each conical hole with a CMM rigid probe and a spherical stylus on the arm extremity. Performance tests are carried out according to ASME B89.4.22, 2004, and the uncertainty of the virtual sphere plate is calculated. As mentioned in [18], the virtual sphere concept is applied to reduce the number of test positions specified in the standards [4,5,6] and increase the efficiency of the verification procedure. Another approach to this concept, in which a virtual circle is used instead of a virtual sphere, is presented by Gonzalez et al. [21,22], where two gauges of anodized aluminum alloy shaped like an inverted T profile and with a length of 1000 mm are manufactured. Four groups of three machined conical holes are used to determine the virtual circles. These four groups use two distances of 500 mm (circles 1 and 2) and 920 mm (circles 3 and 4), respectively. The diameter of the virtual circle does not require calibration because only the dispersion of the measurements is considered.

AACMMs are manually operated, which allows the measurement of a point from multiple arm configurations. This is one of its main advantages. However, AACMMs also have drawbacks in that their repeatability and accuracy are lower than CMMs. To address this point and accurately identify the metrological characteristics of the arm, Cuesta et al. [23,24] develop a new gauge for calibration and verification of AACMMs that incorporates multiple physical geometries in the same gauge. In addition, the gauge includes conical holes machined at its ends that enable the generation of virtual spheres and distance calculations between their centers. In [25], Furutani et al. propose a method to identify the optimal geometric parameters of a measuring arm is described to define the measurement uncertainty of the model. The study also focuses on the type of artifact to be used, depending on the arm’s configuration, and analyzes the minimum number of measurement positions needed to identify the parameters.

There is a wide range of existing approaches to calibration and verification procedures for AACMMs but also a great variety of reference artifacts. In this work, we have developed a new verification procedure for AACMMs with an indexed metrology platform (IMP). A calibrated ball bar gauge is used as a reference artifact, and the testing is based on the evaluation standard ASME B89.4.22-2004. The novelty of the procedure is in the possibility of creating virtual points, virtual gauges and virtual distances in the IMP’s mathematical model, using the measurements from a ball bar gauge carried out with the AACMM assembled on the platform as a reference. In this way, an unlimited number of reference distances may be created. This work aims to optimize the existing calibration and verification procedures.

## 2. Verification Procedure with an Indexed Metrology Platform by Virtual Distances

An indexed metrology platform is proposed by Brau et al. [26] as an auxiliary instrument for use in calibration and verification procedures for portable coordinate measuring instruments. The methodology developed in this work using the indexed metrology platform tries to improve the verification procedures for AACMMs in terms of the testing time and working space required compared to the procedures described in the standards [4,5,6]. This new procedure allows the generation of an unlimited number of virtual reference distances through the platform’s mathematical model, thereby minimizing the number of tests positions required of the physical gauge during verification. Alternative to conventional procedures, the reduction in the testing positions is achieved by settling the calibrated gauge in a fixed position in the AACMM’s working volume and measuring the gauge with the AACMM assembled on the IMP from the six platform rotating positions distributed at 60° each. In this way, when the platform rotates to a new position, which permits the AACMM to measure the same point in the ball bar gauge, a new working volume of the instrument is evaluated.

The design of the platform is based on two hexagonal steel platforms, the upper and lower platforms. The upper, or mobile platform, rotates around the lower platform and is the one on which the portable coordinate measuring instrument is fixed. The lower platform is located in the base of the IMP. The overall platform includes six capacitive sensors and targets with nanometer resolution, which are located in the lower and upper platforms, respectively. These sensors enable the IMP to precisely measure the orientation and position of the upper platform with respect to the lower platform. Three of the sensors are located axially, and the other three are tangentially placed with respect to the rotation axis of the IMP. The capacitive sensor probe model used is a C5-E from vendor Lion Precision, with a measuring range of 100 µm for an output voltage from 10 to −10 V and an operational range from 100 to 200 µm.

Two coordinate reference systems are defined for the upper and lower platforms. Using the capacitive sensor readings, we are able to generate a homogenous transformation matrix (HTM) that links the coordinate reference systems through the mathematical model of the platform [26]. Thus, a point captured with the AACMM and expressed in the arm’s coordinate reference system may be expressed on the lower platform or global coordinate reference system.

An estimation of the measurement uncertainty of the platform was developed in a previous work by the authors [27] using the Monte Carlo method. Similar approaches developing an AACMM uncertainty model using a multi-level Monte Carlo method can be found in [28,29]. In our work, the input variables of the model were first identified, and the *n*-homogeneous transformation matrices (XYZABC) were considered the output variables of the IMP’s mathematical model. Table 1 shows the IMP position and orientation uncertainty for a given platform position and point measured. Two reference calibrated distances were defined, d12 = 100.8024 mm and d15 = 399.9613 mm, in order to calculate the distance error value given as the difference between the distance in the Monte Carlo simulation and the calibrated distance value in the ball bar. The Table 2 presents the uncertainty of the IMP in a distance measurement obtained as a result of running the Monte Carlo simulation over 10,000 iterations.

### 2.1. Kinematic Model Integration

**Figure 1:**
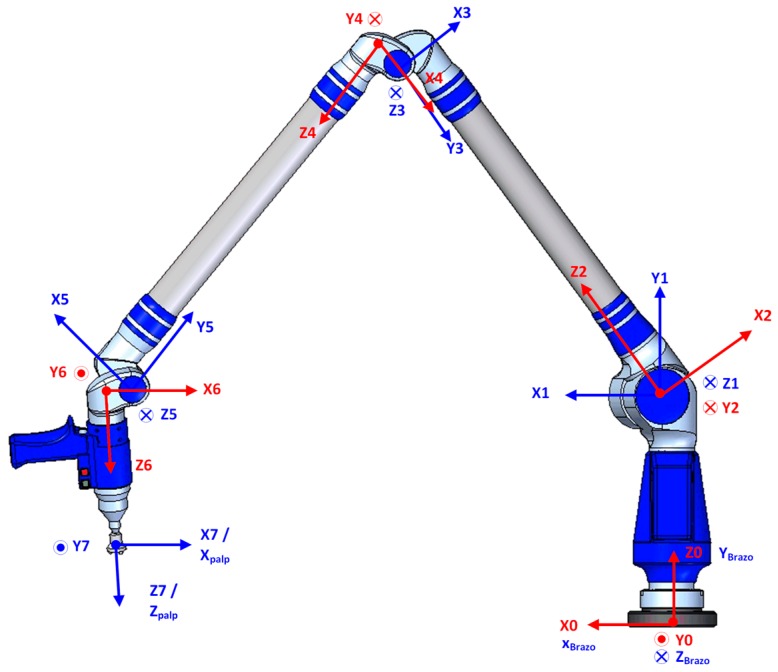
Faro Platinum coordinate reference system in the initial position according to D-H model.

The development of the verification procedure starts with the construction of the kinematic model of the AACMM and the indexed metrology platform, defining the geometric transformations, the location of the coordinate reference systems and the initial nominal geometric parameters of the model. The integration of the AACMM’s kinematics and the platform mathematical model enables the expression of a point captured with the AACMM in the global platform’s coordinate reference system (RS Global) located in the lower platform. By using the mathematical model of the platform, it is possible to calculate a homogenous transformation matrix (HTM) that will allow the change of the coordinate reference systems required. The kinematic model of the AACMM used in this work, Faro Platinum, is based on the Denavit–Hartenberg model (D-H) [30]. Using the D-H model, the coordinates of a point measured with the AACMM in terms of the angles and distance values of the kinematic chain can be obtained. The kinematic model according to the D-H model is shown in Figure 1 and includes a global coordinate reference system (x_0_, y_0_, z_0_) situated on the base of the arm, one coordinate reference system per each rotary joint and the last coordinate reference system located in the stylus (x_7_, y_7_, z_7_) corresponding to the rotation of the wrist of the AACMM.

Once the coordinate reference systems of the model are defined, the next step is to determine the geometric parameters of the model, *di, ai, θi* and *αi*. The initial values of the geometric parameters of the D-H model, i.e., *di, ai, θi* and *αi*, are included in Table 3.

The notation used to express a point measured with the AACMM in the AACMM’s global coordinate reference system with the origin in the arm (x_0_, y_0_, z_0_), initially expressed in the stylus reference system (x_palp_, y_palp_, z_palp_), in terms of *θ*, φ and *d*, is defined by Equation (1):

(1) [XYZ1]x0,y0,z0 (RS0)=T70[XYZ1]Xpal,Ypal,Zpal (RS7)

where ^0^T_7_ is the homogeneous transformation matrix (HTM) expressed in terms of the product of successive coordinate transformation matrixes *^i^*^−1^A*_i_*, *θ* and φ are values obtained from the angular encoders and *d* is the measured distance, as shown in Equation (2):

^0^T_7_ = ^0^A_1_^1^A_2_…^6^A_7_ (2)

**Figure 2:**
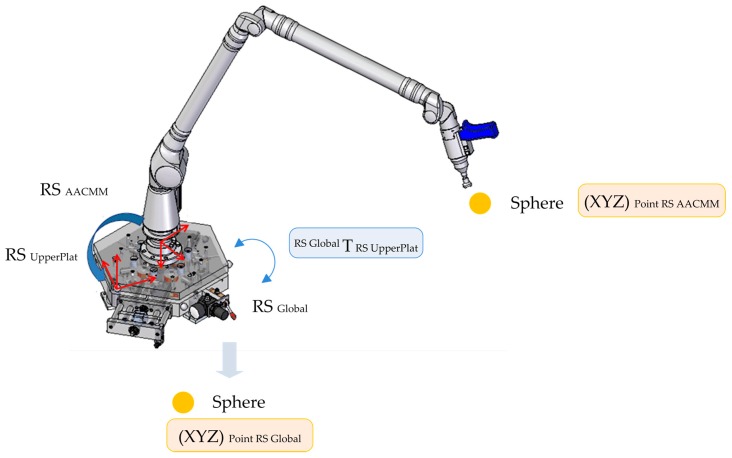
Faro Platinum arm, upper platform and lower platform coordinate reference systems.

The indexed metrology platform model uses the optimum platform’s geometric parameters found during its calibration and the readings from the capacitive sensors captured for each point measured with the AACMM in the verification procedure. For each point measured, a single homogeneous transformation matrix (HTM) is generated. This matrix allows the transformation from the upper platform reference system (RS_UpperPlat_) to the global reference system (RS_Global_) located in the lower platform for the six platform positions. The notation used to express a point measured with the AACMM with its coordinates in the AACMM’s coordinate reference system (RS_AACMM_) into the fixed global coordinate reference system of the lower platform (RS_Global_) for each of the six positions of the platform is simplified and shown in Equation (3) and is calculated via the platform’s mathematical model. This concept is graphically explained in Figure 2.

(3) [XYZ1]RSGlobal=TRSUpperPlatformRSGlobalTRSAACMMRSUpperPlatform[XYZ1]RSAACMM

### 2.2. Verification Procedure Methodology

**Figure 3:**
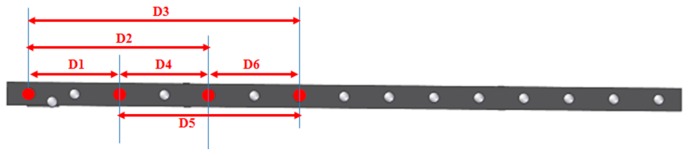
Distances between spheres centers in the gauge, position Diag45 upwards.

The AACMM model used in the testing is a Faro Platinum arm with seven axes, 2.4 m measuring range, a volumetric accuracy of ±0.043 mm and a single point repeatability of 0.030 mm. A ball bar gauge with a length of 1400 mm was selected as the calibrated gauge and was located in different poses in the AACMM’s working volume. The definition of the reference distances to measure together with their distribution in the working volume of the arm was done taking as a reference the volumetric performance evaluation included in the ASME B89.4.22-2004 [4] standard. The following parameters were considered in the definition of the positions: the gauge length (short 800 mm/long 1400 mm), the ball bar gauge inclination (horizontal, vertical and 45°), the ball bar gauge direction with respect to the AACMM (radial or tangential for horizontal dispositions and 45°), the ball bar gauge distance to the center of the working volume, the height of the ball bar and the octants affected. The main target is to test as many possible arm angle combinations as possible in the selected verification testing positions. In this work, a 45° diagonal position tangential to the AACMM, known as Diag45 upwards, which covered two octants of the working volume of the arm, was used as a reference and was measured with the AACMM from all of the platform positions (1–6). Five spheres were measured, capturing nine points per sphere and calculating the distances between the spheres’ centers and their deviations in length versus the calibrated distances, as shown in Figure 3.

The physical gauge is measured with the AACMM assembled on the IMP from all of the rotating positions of the platform (1–6). Each time the platform rotates 60° to a new position, the AACMM measures the same physical gauge from a different platform position, which is equivalent to measuring six physical gauges located in different locations in the AACMM’s working volume from the same platform position. The measurements of the same gauge carried out from the six platform positions define the measured points, resulting in six measured gauges. The Euclidean distances between the points measured are known as measured distances and are used as a parameter in the volumetric performance evaluation of the AACMM. In parallel to the measurement of the points, the values of the capacitive sensors assembled in the indexed metrology platform are captured for each measurement and platform position. These captures are used to obtain a single homogenous transformation matrix per point measured that expresses a point captured by the AACMM in a global coordinate reference system located in the lower platform, as explained in Section 2.1.

**Figure 4:**
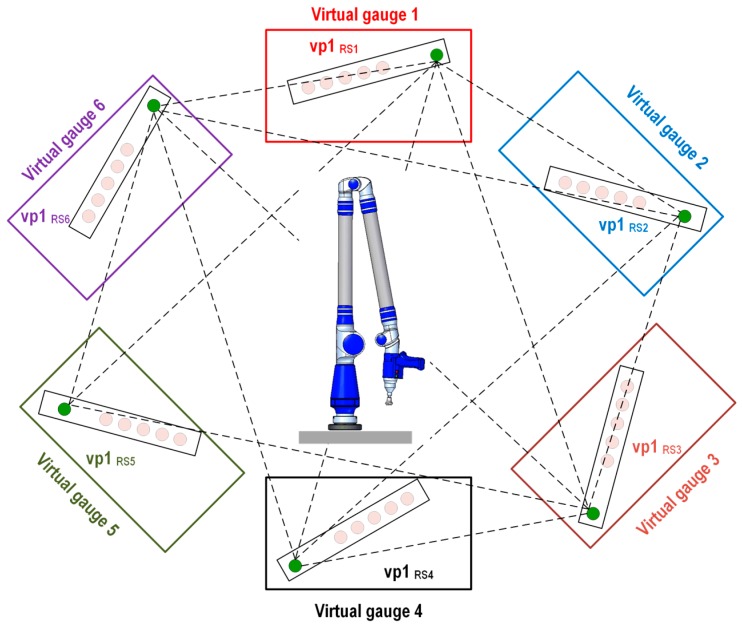
Virtual gauges (1–6).

If we take as a reference the measurements from the gauge done with the AACMM from a selected platform position and because we know with high accuracy the position of the upper platform with respect to the lower platform, the generation of the virtual gauge using the IMP model is based on applying the known rotation angle of the platform to the measured ball bar gauge and being able to generate virtual gauges rotated at 60°/120°/180°/240°/300° from the measured gauge in the selected position of the platform. For example, if we consider the gauge measured in platform position number 1 to be a reference, known as measured gauge 1, it is possible to use the mathematical model of the indexed metrology platform to generate a set of virtual points that will integrate virtual gauge 1, corresponding to the indexed metrology platform’s position number 1. This procedure is repeated successively for the six rotating platform positions, creating the six virtual gauges, as shown in Figure 4. The six virtual gauges are affected by the 60° rotation of the platform from one position to the next. In this work, it is taken as a reference, the coordinate reference system corresponding to platform position 1 which is known as the AACMM reference system 1 (RS_1_). Therefore, a point measured from the AACMM reference system 1 (RS_1_) will have the same coordinates as its equivalent virtual point expressed in the AACMM reference system 1 (RS_1_).

Here, we explain the procedure to generate a virtual point via the mathematical model of the IMP. The procedure starts with the measurement of a point, point 1, on the ball bar gauge with the AACMM assembled on the indexed metrology platform from platform position 1. The coordinates of this point 1 are expressed in AACMM reference system 1, RS_1_. The second step is rotating the platform 60° from position 1 to position 2 and to measure again from this platform position 2, the point 1. The coordinates of this point are now expressed in AACMM reference system 2, RS_2_. We could say that this second measurement is equivalent to measuring a virtual point rotated 60°, known as point 1’ from platform position 1, with its coordinates expressed in AACMM reference system 1, RS_1_. The virtual point is thus affected by the rotation of the platform from platform position 1 to platform position 2.

Taking as a reference one point measured using platform position 1, where the measured and virtual points have the same coordinates in the virtual gauge 1 and expressing these coordinates in AACMM reference system 1 (RS_1_), it is possible to generate a virtual point in virtual gauge 2 through the indexed metrology platform’s mathematical model. In this calculation, the translational and rotational components of the homogeneous transformation matrix, which changes from platform position 1 to platform position 2, are considered, assuming that the coordinates of the virtual point are expressed in the AACMM reference system 1 (RS_1_), as shown in Equation (4).

(4) [XYZ1]RS2=TRS1RS2[XYZ1]RS1

**Figure 5:**
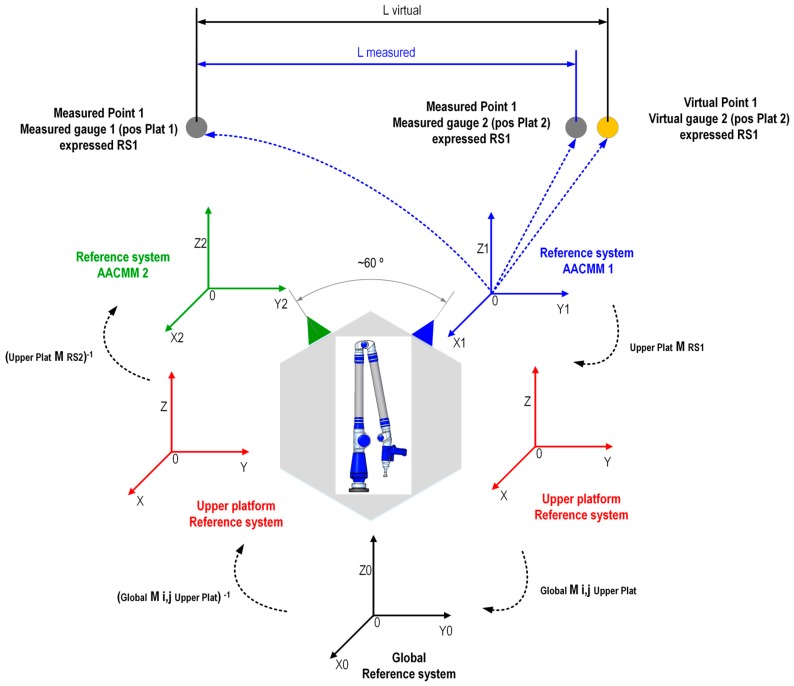
Measured distance and virtual distance concept.

The T matrix is a homogeneous transformation matrix that provides a change in the coordinates from AACMM reference system 1 (RS_1_), corresponding to the virtual point in virtual gauge 1, to AACMM reference system 2 (RS_2_)_,_ where the new virtual point in virtual gauge 2 is created by the rotation of the platform. The main difference is the assumption that the new virtual point generated in virtual gauge 2 will have its coordinates expressed in the AACMM reference system 1 (RS_1_), as though the AACMM were measured from position 1 of the platform, as explained in Figure 5.

The homogeneous transformation matrix T is expressed in Equation (5),

(5) TRS1RS2=(MRS2RSUpperPlat)-1(Mi,jRSUpperPlatRSGlobal)-1Mi,jRSUpperPlatRSGlobalMRS1RSUpperPlat

where

- MRS2RSUpperPlat: AACMM reference system 2 to upper platform reference system homogeneous transformation matrix.
- MRS1RSUpperPlat: AACMM reference system 1 to upper platform reference system homogeneous transformation matrix.
- Mi,jRSUpperPlatRSGlobal: Upper platform reference system to global platform reference system homogeneous transformation matrix. This matrix is generated per each measured point out of the values of the capacitive sensors assembled in the indexed metrology platform.

The next concept to be developed is the generation of the virtual distance between the virtual points. With this procedure, it is possible to create an unlimited number of Euclidean distances of different lengths between virtual points located either in the same or in different virtual gauges. Taking platform position 1 as a reference, the concepts of measured distance and virtual distance are shown in Figure 5. The measured distance is defined as the Euclidean distance between point 1 measured from platform position 2, assuming that its coordinates are expressed in AACMM reference system 1 (RS_1_), and the coordinates measured from platform position 1 and expressed in AACMM reference system 1 (RS_1_). The virtual distance is defined as the Euclidean distance between virtual point 1 generated in virtual gauge 2, assuming that its coordinates are expressed in AACMM reference system 1 (RS_1_), and virtual point 1, with its coordinates expressed in AACMM reference system 1 (RS_1_). It is necessary to note that the coordinates of virtual point 1 in virtual gauge 2 and the coordinates of measured point 1 from platform position 2, both of which are expressed in AACMM reference system 1 (RS_1_), will not be exactly equal due to the error in the indexed metrology platform. This deviation in the coordinates between the measured and virtual points is translated into a distance error D_i_ between the virtual distance L_Virtual_ and the measured distance L_Measured_.

To evaluate the volumetric performance of the AACMM in its working volume using the virtual distances method, three parameters are selected. First, we examine the maximum distance error among all positions of the platform, the range of the distance errors and a mean distance error. Four evaluation alternatives using the virtual distance technique were developed in this work and are explained, considering AACMM reference system 1 (RS_1_) to be the reference system for the procedure.

#### 2.2.1. Evaluation Method 1: Virtual Distances among Virtual Points in Gauge 1 and Equivalent Virtual Points in Gauges (2–6)

**Figure 6:**
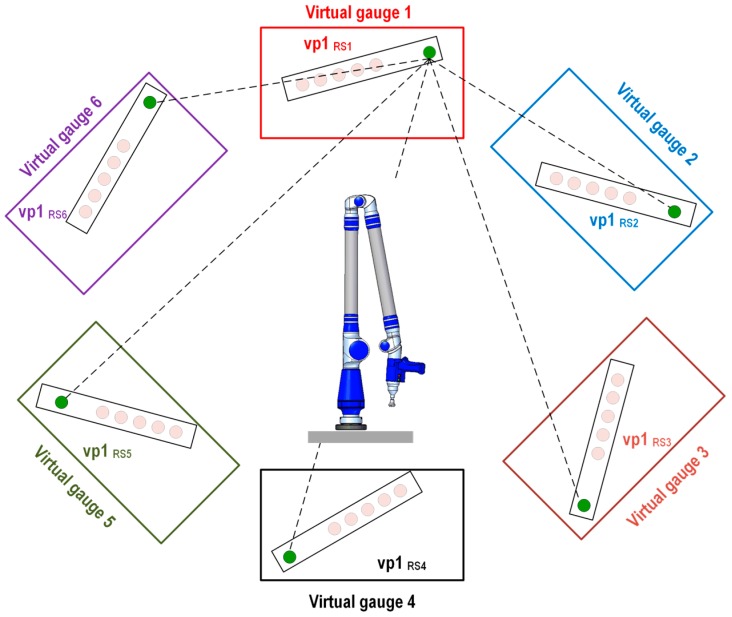
Evaluation method 1: virtual distance between virtual point in gauge 1 and equivalent virtual points in virtual gauges (2–6).

The first evaluation method targets the definition of a virtual distance based on distance calculations among the center coordinates of the four virtual balls in virtual gauge 1 and their coordinates in the other virtual gauges (2–6). This method enables the generation of virtual distances of different lengths that are larger and greater in number than those that can be defined using a physical ball bar gauge. A total of 20 virtual distances are calculated, as shown in Figure 6, with their graphical representations shown as six example virtual points 1, vp1_RS1_–vp1_RS6_, generated in the corresponding virtual gauges (1–6). The Euclidean distance between virtual point 1 (vp1_RS1_) located in virtual gauge 1 is used as a reference. The remainder of the virtual points (vp1_RS2_–vp1_RS6_) generated in virtual gauges 2 to 6 may be obtained using Equation (6):

(6) $$D_{i,j}=\sqrt{{\left(X_{i,j}-X_{1,j}\right)}^{2}+{\left(Y_{i,j}-Y_{1,j}\right)}^{2}+{\left(Z_{i,j}-Z_{1,j}\right)}^{2}}\;i=2,\ldots ,6;\;j=1,\ldots ,4$$

where *D_i,j_* is the Euclidean distance between the virtual point *j* in each of the *i* virtual gauges, and the virtual point *j* in virtual gauge 1 with their coordinates expressed in AACMM reference system 1 (RS_1_).

#### 2.2.2. Evaluation Method 2: Virtual Hexagon, Evaluation through Virtual Distances among Virtual Points in Consecutive Gauges

**Figure 7:**
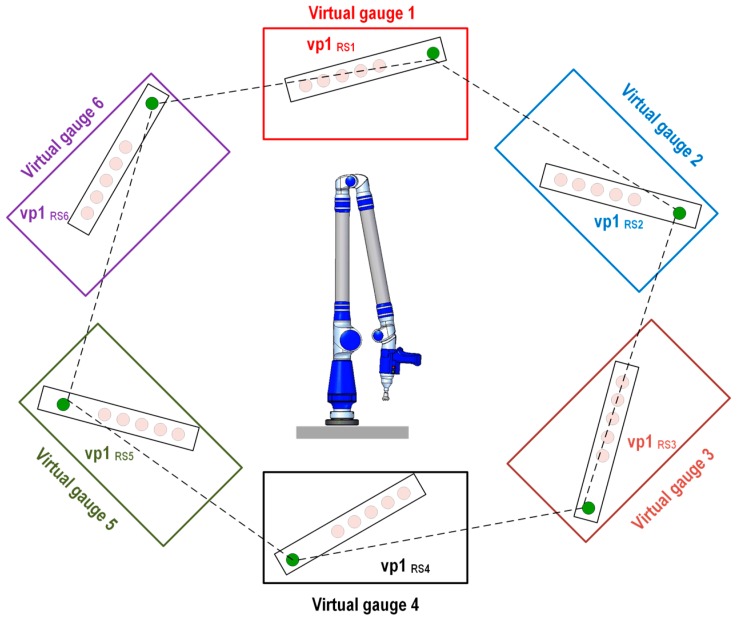
Evaluation method 2: virtual distance between virtual point in gauge 1 and their equivalent virtual points in consecutive gauges.

The virtual distances hexagon method focuses on evaluating the instrument’s error at different heights and rotation angles because of the platform’s rotation. Four virtual hexagons are generated depending on the virtual point’s height in the virtual gauge. The definition of the virtual distance is based on the generation of virtual distances between the coordinates of the equivalent virtual balls’ centers located in consecutive gauges, which define the virtual hexagons at different heights. A total of 20 virtual distances are defined, and a representation of the virtual distances generated in the six virtual gauges considering virtual point 1 (vp1) as an example is shown in Figure 7. The Euclidean distances between the virtual points situated in virtual gauge *i* and the equivalent virtual points located in the consecutive virtual gauges according to the positions of the platform (1–6) are shown in Equation (7):

(7) $$D_{i,j}=\sqrt{{\left(X_{i+1,j}-X_{i,j}\right)}^{2}+{\left(Y_{i+1,j}-Y_{i,j}\right)}^{2}+{\left(Z_{i+1,j}-Z_{i,j}\right)}^{2}}\;i=1,\ldots ,5;\;j=1,\ldots ,4$$

where *D_i,j_* is the Euclidean distance between the virtual point *j* in each of the *i* virtual gauges, and the virtual point *j* in the next virtual gauge *i* + 1, with their coordinates expressed in AACMM reference system 1 (RS_1_).

#### 2.2.3. Evaluation Method 3: Evaluation through Crossed Virtual Distances among Virtual Points in Different Virtual Gauges

**Figure 8:**
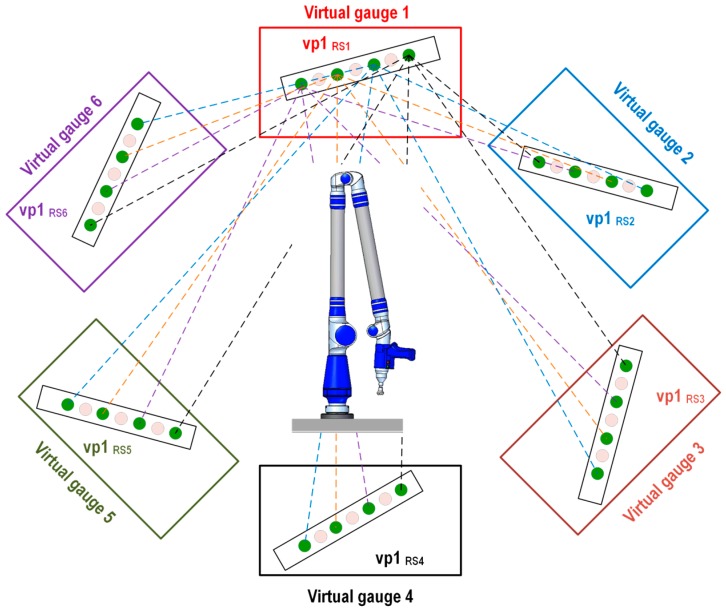
Evaluation method 3: crossed virtual distances between virtual gauge 1 and virtual gauges (2–6).

The following evaluation method is based on the generation of crossed virtual distances among virtual balls located in the six virtual gauges. In this way, 20 crossed distances among the four different spheres in different virtual gauges are evaluated, as shown in Figure 8. The Euclidean distances between virtual points located in virtual gauge 1, which is used as a reference, and the rest of the virtual points generated in virtual gauges (2–6) according to the positions of the platform are listed in Table 4.

#### 2.2.4. Evaluation Method 4: Evaluation by Horizontal, Vertical and Diagonal Virtual Distances Using Virtual Gauges (1–6)

**Figure 9:**
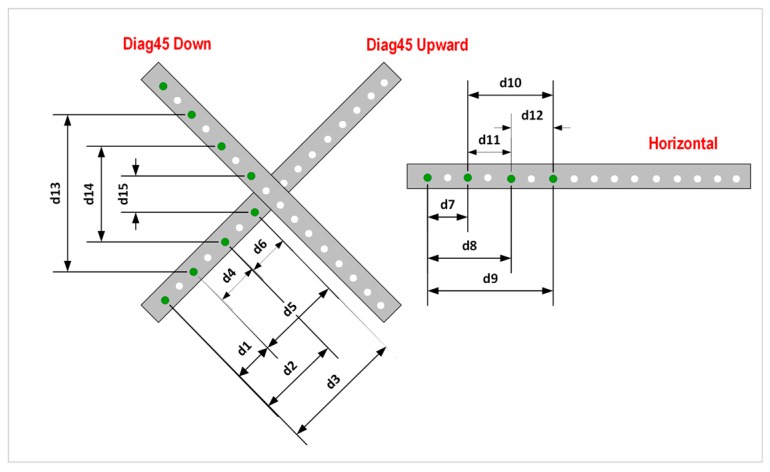
Evaluation method 4: virtual vertical, horizontal and diagonal distances in virtual mesh 1.

The last evaluation method is based on virtual distances defined among virtual balls located in the same virtual gauge or mesh of virtual points. Two new ball bar gauge measured positions were included in this method, following the recommendations in the standards [4,5,6]: one additional diagonal position at a 45° inclination (Diag45 down) and a horizontal position (Horizontal). With these two new poses, we gain flexibility in the distance definition, which permits the generation of horizontal, vertical and diagonal virtual distances among the spheres. From these, 15 virtual distances classified according to their position (horizontal, vertical or diagonal) are created, as shown in Table 5 and Figure 9. Virtual gauge 1 is taken as an example, but the same virtual distances are replicated in virtual gauges 2 to 6, which are used to generate the complete virtual distance scenario for this evaluation method.

## 3. Results of the AACMM Verification Procedure with the Virtual Distances Method

The results obtained in the verification of the AACMM by applying the four virtual distance evaluation methods developed in this work are shown in Table 6 and exhibit an average distance error of 0.0466 mm. The maximum distance error corresponds to evaluation method 2, virtual hexagon, with a maximum distance error value of 0.1291 mm. The mean range of the deviations is 0.1016 mm.

Some differences in the results have been identified among the four evaluation methods due to their different approaches to the definition of the virtual distances. Methods 1 and 3 show comparable results by evaluating the longest distances, as seen in Table 7. This is because the virtual distance can be defined in these methods among the points in virtual gauge 1 and the points located in virtual gauges (2–6). In this way, the virtual distances are greater than those that can be evaluated in a 1400 mm physical ball bar gauge. Method 2 presents the maximum distance error value (0.1291 mm), which indicates that this method may be more sensitive to the platform’s azimuthal rotation error (θ) because the virtual distances are generated between the coordinates of the virtual points located in consecutive meshes. Its mean distance error value of 0.0784 mm is also the highest among the methods. The lowest mean distance error value (0.0186 mm) is obtained using method 4: this measurement error is local to the mesh because the distances are always calculated among spheres located in the same virtual mesh. Additionally, the lengths evaluated in method 4 are smaller than those in methods 1–3, which cover a range from 200 mm to 749 mm. This factor influences the distance error result.

**Figure 10:**
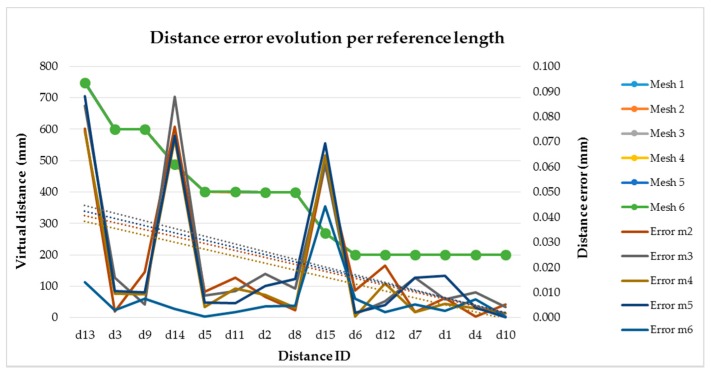
Distance error per virtual distance evaluated (evaluation method 4).

Focusing on evaluation method 4, it is possible to analyze the influence of the measuring length magnitude on the measurement error. In this case, based on the virtual distances or virtual reference lengths described in Figure 9, the distance error increases with the reference length dimension. In Figure 10 we represent for each mesh (1–6) the virtual reference lengths (y axis) listed in Table 5 sorted in the figure in decreasing magnitude value. In this case, mesh 1 to mesh 5 distances are overlapped with mesh 6 distances, in green in the graphical representation, due to the small differences detected among the meshes. Then, the distance errors (y’ axis) are calculated for each mesh (2–6) as the difference between the measured and virtual distances. In mesh 1, measured and virtual distance are the same due to the selection of the AACMM reference system 1 (RS_1_) as a reference for the procedure. As seen in Figure 10 a decreasing trend for the distance error is observed when the distance length decreases for all the virtual meshes (2–6). It should also be mentioned that higher values of the distance error are detected for the virtual reference lengths d13 (0.088 mm), d14 (0.088 mm) and d15 (0.069 mm) corresponding to virtual distances generated among spheres located in two different gauge poses as shown in Figure 9.

**Figure 11:**
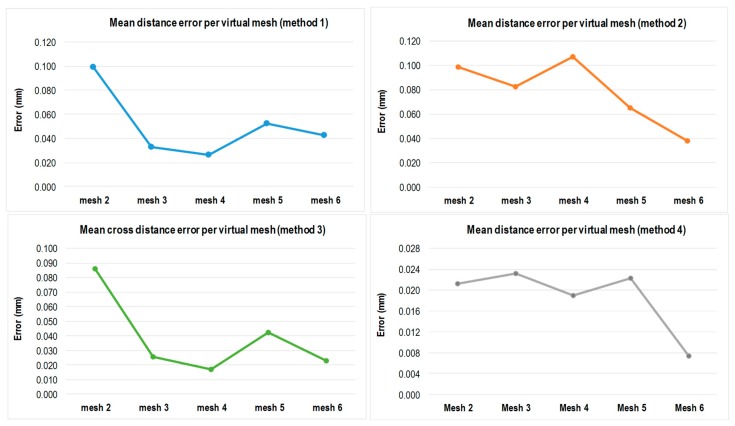
Mean distance error per virtual mesh.

Another aspect to consider when assessing the behavior of the IMP in terms of its rotating positions is the mean error value per virtual mesh, as shown in Figure 11 for all the evaluation methods. Methods 1 and 3 exhibit similar tendencies with higher mean error values in mesh 2, 0.0990 mm and 0.0860 mm, respectively, and these values decrease for the rest of the meshes. For method 2, the virtual hexagon, the mean error reaches its maximum in mesh 4 (0.107 mm), corresponding to a 180° rotation of the platform.

As previously explained, this work develops a new methodology for evaluating AACMMs with an indexed metrology platform, thus presenting the possibility of generating an unlimited number of virtual reference lengths by using the IMP’s mathematical model. As a result, the physical gauge could be partially substituted after its first measurement because the platform itself provides the possibility to generate virtual gauges and virtual reference lengths from this initial measurement with the gauge. It is necessary to compare the results obtained in the verification procedures developed with the indexed metrology platform using the virtual distances concept and without the virtual distances. The verification procedure for AACMMs using the IMP without virtual distances uses a physical ball bar gauge located in different positions of the working volume of the arm. The AACMM is placed on the IMP, which allows the rotation of the AACMM into six different rotating positions, to measure with the arm the ball bar gauge from all the platform’s positions. When the AACMM measures the same sphere on the gauge from a different platform’s position, a new instrument working volume is evaluated. This is therefore equivalent to measuring a new gauge physical position. The platform itself allows an increase in the number of test positions of the gauge without needing to define a new pose during testing. The virtual distances methodology goes further in that it is able to not only generate a new gauge’s poses for each position of the platform but also broaden the number and type of reference distances defined among the new virtual points.

Taking as a reference the measurements carried out in the Diag45 upward gauge position, the mean and maximum values for the distance errors obtained in both the AACMM’s verification procedures using the IMP with and without the virtual distances methodology are listed in Table 8. The distance error is calculated in the verification procedure with the IMP as the difference between the measurement on the gauge and the calibrated distance. In the case of the virtual distances methodology, there is no existing physical calibrated gauge for all the reference distances. Therefore, the reference artifact is the virtual gauge itself, which is synthetically generated through the IMP’s mathematical model.

In general, the virtual distances method demonstrates higher mean, maximum and 2RMS error values than did the verification procedure without virtual distances, as shown in Table 8. This could be realistic because, as previously assessed, the measurement error of the AACMM with the IMP increases with the reference length’s magnitude and because the virtual distances method tests longer reference lengths of up to 1696 mm. Meanwhile, using the verification method without virtual distances, the maximum reference lengths evaluated are up to 800 mm.

We can also analyze the distance reproducibility per platform position, which is calculated from the difference between the virtual and measured distances for platform position 1 and the results obtained for the rest of the platform positions (2–6). The mean and maximum values of the 30 distance errors obtained for the Diag45 upwards gauge position are listed in Table 9. The mean distance reproducibility error values obtained with and without virtual distances are both 0.0070 mm, which confirms that under these testing conditions, the virtual distance methodology is suitable for the evaluation’s requirements. The maximum distance reproducibility errors were 0.0174 mm and 0.0229 mm, respectively.

## 4. Conclusions

This work defines a new procedure for the verification of AACMMs together with a capacitive sensor-based indexed metrology platform. These sensors assembled in the platform enable the IMP to precisely measure the orientation and position of the upper platform with respect to the lower platform. Consequently, using the capacitive sensor readings, we are able to generate a homogenous transformation matrix that links the coordinate reference systems through the mathematical model of the platform. Thus, a point captured with the AACMM and expressed in the arm’s coordinate reference system may be expressed on the lower platform or global coordinate reference system.

The new verification procedure is based on the use of the platform to synthetically generate virtual points and distances using the mathematical model of the platform. These virtual distances are used as a reference length in the verification procedure for the arm and replacing the physical gauge. The main advantage of the new procedure is the unlimited number and types of reference distances that can be created without the need to use physical reference artifacts. The IMP is used as an auxiliary instrument on the mounting arm which rotates jointly with the platform into its six rotating positions, which are defined at 60° each. Using the measurements carried out on a ball bar gauge from all positions of the platform as a reference, it is possible to create virtual gauges in different positions and orientations in the working volume of the AACMM by applying the known rotational angle of the platform to the gauge. Six virtual meshes are created with their corresponding virtual ball bar gauges, and four evaluation methods are used to generate the virtual reference distances defined in the procedure. This new methodology is not only suitable for AACMMs but also for high-range measuring instruments, where the required reference distances are longer and the reference artifacts and testing facilities are usually more complex. The results obtained in the four evaluation methods described in the manuscript have an average mean distance error of 0.0466 mm and a maximum distance error of 0.1291 mm. A clear relationship between the distance error and the magnitude of the reference distance evaluated in all of the virtual meshes was also shown. In addition, the mean distance error value obtained using the virtual distances (0.0466 mm) is reasonably close to the MPE (maximum permissible error) of the arm according to the manufacturer data for volumetric evaluation (±0.043 mm), and the error of the IMP in a distance measurement is 0.245 µm, as assessed by developing the IMP’s uncertainty model.

To validate the new procedure using virtual distances, we compared the results obtained in the verification of the same AACMM with the IMP without using the virtual distances methodology. The mean distance error (0.0203 mm), 2RMS (0.0591 mm) and maximum distance error (0.0902 mm) values obtained in the verification of the AACMM with the IMP were compared to the virtual distances procedure’s results. The errors in the virtual distances procedure are higher than in the procedure without virtual distances. In the virtual distance methodology, the reference distances evaluated are longer due to the wider range of possibilities offered in the distance definition process, which could have a direct influence on the distance error, as shown in our testing.

In summary, we conclude that the virtual distances methodology used in the volumetric evaluation of AACMMs with the IMP produces comparable results to the verification procedure using the IMP without the virtual distances method. As a result, we can assess that the virtual methodology with the IMP is an adequate alternative procedure for evaluating AACMMs by accounting for the required accuracy of the measuring instrument. This results in a significant reduction in the number of measurements and the amount of time required in a new verification procedure.

## Figures and Tables

**Table 1:** Indexed metrology platform position and orientation uncertainty in homogeneous transformation matrices upper to lower platform, sphere 1, point 1, 10,000 iterations.

^RS_Global_^T_RS_UpperPlat__ (Sphere 1/Point 1/Platform Position 1)			
	Nominal	Mean	Uncertainty (µm or °)
X (mm)	−0.13500	−0.13502	0.020
Y (mm)	196.61710	196.61707	0.045
Z (mm)	40.84180	40.84181	0.050
A (°)	179.99880	179.99879	0.020
B (°)	0.01940	0.01941	0.017
C (°)	60.05620	60.05621	0.012

**Table 2:** Indexed metrology platform uncertainty in a distance measurement, 10,000 iterations.

	d12 (Sphere 1–2)	d15 (Sphere 1–5)
Mean distance error (µm)	58.721	63.400
Standard deviation (µm)	0.245	0.242

**Table 3:** Initial values of the arm D-H model geometric parameters.

Joint	*θi* (°)	*αi* (°)	*ai* (mm)	*di* (mm)
1	0	90	50	75
2	135	90	0	0
3	0	−90	30	590
4	90	−90	30	0
5	180	−90	30	590
6	135	−90	30	0
7	0	0	0	215

**Table 4:** Evaluation method 3: crossed virtual distances definition.

Distance	Sphere Number (Virtual Gauge 1)	Sphere Number (Virtual Gauges (2–6))
1	1	3
2	3	5
3	5	7
4	7	1

**Table 5:** Evaluation method 4: Virtual distances definition by mesh (1–6).

Distance	Virtual Ball A	Virtual Ball B	Distance Type	Gauge Position
1	1	3	Diagonal	Diag45 upwards
2	1	5	Diagonal	Diag45 upwards
3	1	7	Diagonal	Diag45 upwards
4	3	5	Diagonal	Diag45 upwards
5	3	7	Diagonal	Diag45 upwards
6	5	7	Diagonal	Diag45 upwards
7	1	3	Horizontal	Horizontal
8	1	5	Horizontal	Horizontal
9	1	7	Horizontal	Horizontal
10	3	5	Horizontal	Horizontal
11	3	7	Horizontal	Horizontal
12	5	7	Horizontal	Horizontal
13	3	3	Vertical	Diag45 down–Diag45 upwards
14	5	5	Vertical	Diag45 down–Diag45 upwards
15	7	7	Vertical	Diag45 down–Diag45 upwards

**Table 6:** Distance error results per virtual distances evaluation method.

	Method 1	Method 2	Method 3	Method 4	Mean	Maximum
Mean distance error (mm)	0.0507	0.0784	0.0386	0.0186	0.0466	
Max distance error (mm)	0.1132	0.1291	0.1004	0.0882		0.1291
Range of distance error (mm)	0.1072	0.1126	0.0985	0.0879	0.1016	
Standard deviation (mm)	0.0315	0.0283	0.0294	0.0253		

**Table 7:** Reference lengths per method.

	Method 1	Method 2	Method 3	Method 4
Minimum length (mm)	596	596	537	200
Maximum length (mm)	1696	848	1598	749

**Table 8:** Distance error results in AACMM verification with IMP (with and without virtual distances).

	IMP Virtual-Method 1	IMP Virtual-Method 2	IMP Virtual-Method 3	IMP
Mean distance error (mm)	0.0507	0.0784	0.0386	0.0203
Max distance error (mm)	0.1132	0.1291	0.1004	0.0902
2RMS	0.1185	0.1662	0.0961	0.0591

**Table 9:** Distance reproducibility errors in AACMM verification procedure with IMP (with and without virtual distances).

	IMP—With Virtual Distances	IMP—Without Virtual Distances
Mean distance reproducibility error (mm)	0.0070	0.0070
Max distance reproducibility error (mm)	0.0174	0.0229
